# Pilot Study of ^64^Cu(I) for PET Imaging of Melanoma

**DOI:** 10.1038/s41598-017-02691-3

**Published:** 2017-05-31

**Authors:** Lei Jiang, Yingfeng Tu, Xiang Hu, Ande Bao, Hao Chen, Xiaowei Ma, Tim Doyle, Hongcheng Shi, Zhen Cheng

**Affiliations:** 1 Department of Nuclear Medicine, Zhongshan Hospital, Fudan University, 180 Fenglin Road, Shanghai, 200032 China; 2 Molecular Imaging Program at Stanford (MIPS), Department of Radiology and Bio-X Program, Canary Center at Stanford for Cancer Early Detection, Stanford University, Stanford, CA 94305 USA; 3 Department of Radiation Oncology, Case Western Reserve University, University Hospitals, Case Medical Center, 11100 Euclid Ave, Cleveland, OH 44106 USA

## Abstract

At present, ^64^Cu(II) labeled tracers including ^64^CuCl_2_ have been widely applied in the research of molecular imaging and therapy. Human copper transporter 1 (hCTR1) is the major high affinity copper influx transporter in mammalian cells, and specially responsible for the transportation of Cu(I) not Cu(II). Thus, we investigated the feasible application of ^64^Cu(I) for PET imaging. ^64^Cu(II) was reduced to ^64^Cu(I) with the existence of sodium L-ascorbate, DL-Dithiothreitol or cysteine. Cell uptake and efflux assay was investigated using B16F10 and A375 cell lines, respectively. Small animal PET and biodistribution studies were performed in both B16F10 and A375 tumor-bearing mice. Compared with ^64^Cu(II), ^64^Cu(I) exhibited higher cellular uptake by melanoma, which testified CTR1 specially influx of Cu(I). However, due to oxidation reaction *in vivo*, no significant difference between ^64^Cu(I) and ^64^Cu(II) was observed through PET images and biodistribution. Additionally, radiation absorbed doses for major tissues of human were calculated based on the mouse biodistribution. Radiodosimetry calculations for ^64/67^Cu(I) and ^64/67^Cu(II) were similar, which suggested that although melanoma were with high radiation absorbed doses, high radioactivity accumulation by liver and kidney should be noticed for the further application. Thus, ^64^Cu(I) should be further studied to evaluate it as a PET imaging radiotracer.

## Introduction

Copper (Cu) is an essential micronutrient required for many biological processes *in vivo*, such as respiration, iron transport, oxidative stress protection, peptide hormone production, pigmentation, blood clotting, and normal cell growth and development^1–4^. Although Cu is not always abundant in the *in vivo* environment, cells have evolved a complex system of Cu transporters and chaperones that accumulate Cu^3^. Members of copper transporter (CTR) family have been reported to be the copper uptake machinery. Human copper transporter 1 (CTR1), a 190-amino acid protein of 28 kDa with three transmembrane domains, mainly acts as a copper transporter in mammal^5, 6^. Copper metabolism has also been known to be critical for cell proliferation, angiogenesis, and tumor growth^7–9^. CTR1 has been proven to be overexpressed in many types of cancer cells, including melanoma, prostate cancer, liver cancer, and non-small cell lung cancer (NSCLC)^7, 10–12^. Therefore, copper metabolism has been explored as an imaging biomarker for tumor detection^10, 12, 13^.

Copper radionuclides, including ^60^Cu, ^61^Cu, ^62^Cu, ^64^Cu, and ^67^Cu, offer versatile choices for applications in imaging and therapy^14–16^. The short-lived ^60^Cu (t_1/2_ = 23.4 min), ^61^Cu (t_1/2_ = 3.32 h) and ^62^Cu (t_1/2_ = 9.76 min) decay by electron capture and β^+^ emission, and they have been used as to prepare perfusion agents such as Cu-pyruvaldehyde bis(N^4^-methylthiosemicarbazone) (PTSM) and Cu-ethylglyoxal bis(thiosemicarbazone) ETS^17, 18^. The longer-lived ^67^Cu (t_1/2_ = 62.01 h) decays exclusively by β^−^ emission and has been used to label monoclonal antibodies and antibody fragments for radioimmunotherapy^16, 19^. Interestingly, ^64^Cu has an intermediate half-life of 12.7 h and unique decay prolife (β^+^: 18%, β^−^: 38%, and electron capture: 44%), making it a favorable option for radiolabeling nanoparticles, antibodies, antibody fragments, peptides, and small molecules for PET imaging and radionuclide therapy^20, 21^.

Copper is redox-active, and both cupric (II) and cuprous (I) oxidation states are relevant in the biological systems. Of the different oxidation states, Cu(II) is the most common one presenting in ^64^Cu radiopharmaceuticals. ^64^CuCl_2_ has been reported as a promising PET probe for imaging liver cancer, prostate cancer, melanoma, etc.^10, 12, 22^. However, more recently, with the better understanding the role of CTR1 in tumor biology, CTR1 has been found to mainly and specifically transport Cu(I) instead of Cu(II)^3, 23^. Therefore, we hypothesize that ^64^Cu(I) could serve as a novel and promising probe for tumor PET imaging and therapy. In this study, ^64^Cu(I)Cl was prepared by reduction of ^64^Cu(II)Cl_2_, and it was then evaluated *in vitro* and *in vivo* in both B16F10 and A375 tumor models which are known for their high CTR1 expression levels^12^.

## Results

### Cell Uptake

**Figure 1:**
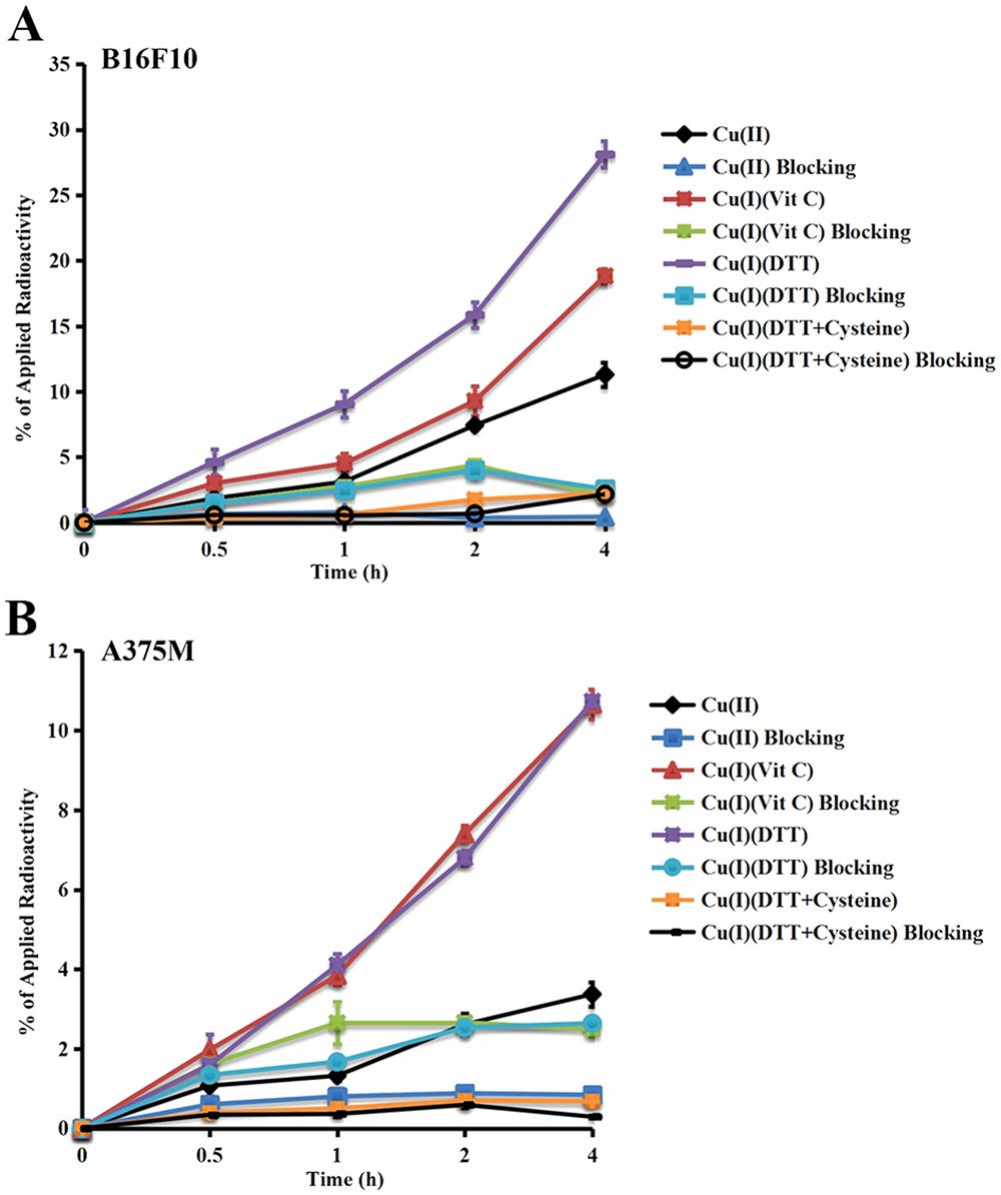
Cell uptake assay of ^64^Cu(II) and ^64^Cu(I) by B16F10 and A375M cells at 0.5, 1, 2, and 4 h, respectively.

As displayed in Fig. 1A, during 4 h incubation period at 37 °C, ^64^CuCl_2_ exhibited the steadily increasing accumulation by B16F10 cells over the observation period, and the maximum uptake was seen at 4 h within the follow up time. The B16F10 cell uptake values of ^64^CuCl_2_ at 0.5, 1, 2 and 4 h at 37 °C were 1.86 ± 0.09%, 3.13 ± 0.82%, 7.43 ± 0.36%, and 11.30 ± 0.91%, respectively. In comparison, the B16F10 cell uptake trend and performance of ^64^CuCl (prepared by ^64^CuCl_2_ with VitC or DTT) was similar to that of ^64^CuCl_2_ but with 1.5–2 folds increasing uptake of the radioactivity. The B16F10 cells uptake values of ^64^CuCl (prepared by ^64^CuCl_2_ with VitC) were 3.01 ± 0.36%, 4.52 ± 0.75%, 9.29 ± 1.13%, and 18.83 ± 0.56%, and of ^64^CuCl (prepared by ^64^CuCl_2_ with DTT) were 4.63 ± 0.43%, 9.06 ± 0.97%, 15.86 ± 2.24%, and 28.11 ± 0.23%, respectively. Interestingly, for the B16F10 cells incubation with ^64^CuCl prepared by ^64^CuCl_2_ with both DTT and cysteine the radioactivity uptake is much lower, and the values at 0.5, 1, 2 and 4 h were 0.37 ± 0.03%, 0.60 ± 0.08%, 1.75 ± 0.06%, and 2.22 ± 0.39%, respectively. There was significant difference among above four groups (*P* < 0.05).

The A375M cell uptake results of ^64^CuCl_2_ and ^64^CuCl were shown in Fig. 1B. ^64^CuCl_2_, ^64^CuCl prepared by ^64^CuCl_2_ with VitC, and ^64^CuCl prepared by ^64^CuCl_2_ with DTT exhibited rapid A375M cell accumulation at the first 30 min, followed by a steady increasing, and the highest uptake was also achieved at 4 h, reaching 3.37 ± 0.03%, 10.66 ± 0.37%, and 10.73 ± 0.04%, respectively. There was significant difference between ^64^CuCl_2_ and ^64^CuCl (*P* < 0.05). Similarly, for the A375M cells incubation with ^64^CuCl prepared by ^64^CuCl_2_ with both DTT and cysteine, the radioactivity accumulation is low, and the values at 0.5, 1, 2 and 4 h were only 0.39 ± 0.04%, 0.50 ± 0.06%, 0.70 ± 0.06%, and 0.68 ± 0.04%, respectively.

For the blocking experiments in either B16F10 or A375M cells, the uptake of ^64^CuCl_2_, ^64^CuCl prepared by ^64^CuCl_2_ with VitC, and ^64^CuCl prepared by ^64^CuCl_2_ with DTT, was significantly lower than the corresponding non-blocking groups at each time point (*P* < 0.05) (Fig. 1A and B). However, there was no significant difference between the blocking and non-blocking groups of ^64^CuCl prepared by ^64^CuCl_2_ with both DTT and cysteine.

### Cell Efflux

**Figure 2:**
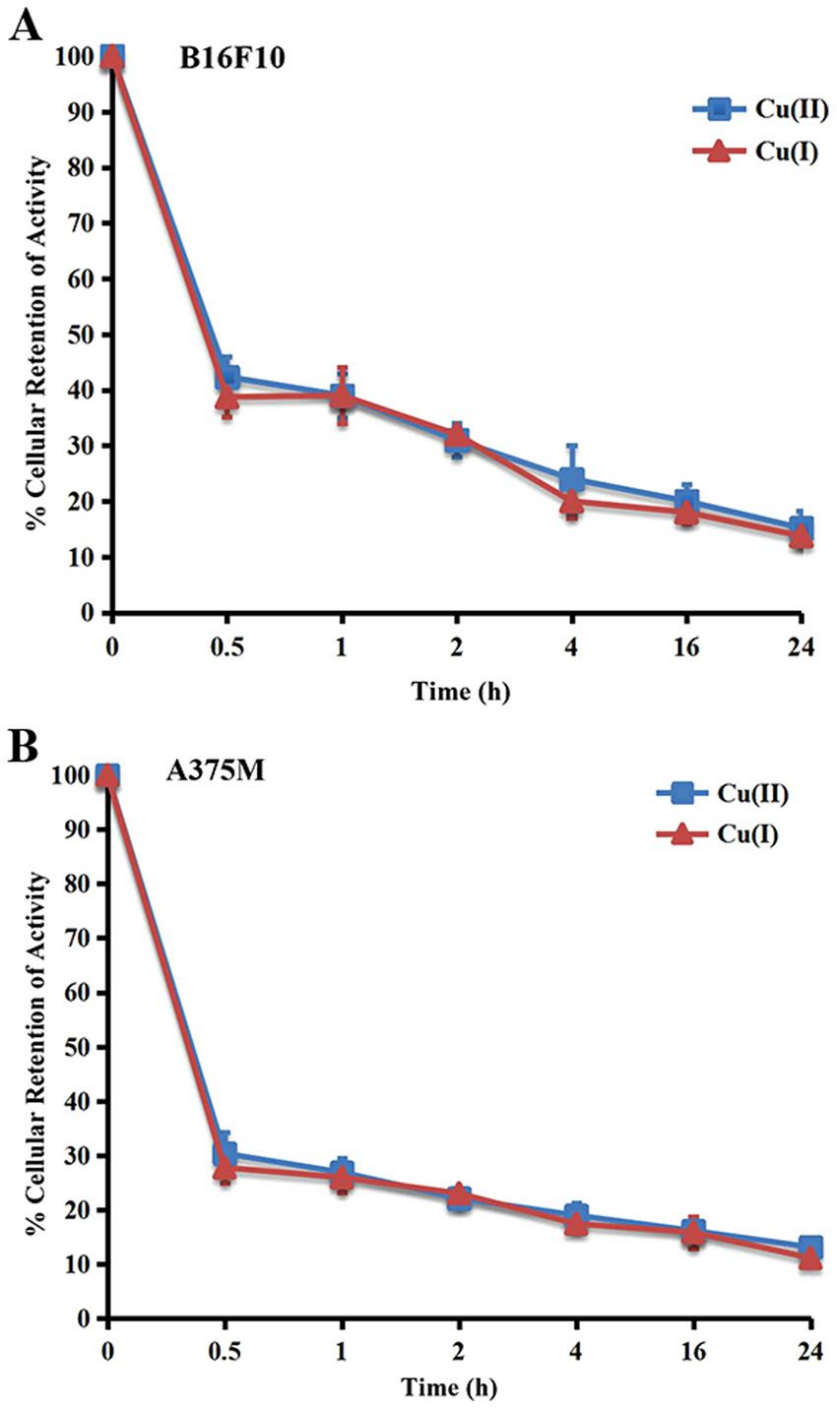
Cell efflux assay from B16F10 and A375M cells after 2 h incubation with ^64^Cu(II) and ^64^Cu(I) at 0.5, 1, 2, 4, 16 and 24 h, respectively.

The radioactivity of ^64^CuCl_2_ and ^64^CuCl prepared by ^64^CuCl_2_ with VitC groups was quickly cleared from both B16F10 and A375M cells. As displayed in Fig. 2A, over a 0.5 h incubation period, 42.36 ± 3.56% of ^64^CuCl_2_ and 38.72 ± 3.59% of ^64^CuCl presented in B16F10 cells, respectively. At 24 h, only 15.16 ± 3.04% of ^64^CuCl2 and 13.74 ± 1.74% of ^64^CuCl were remained (Fig. 2A). Similar to the cell efflux studies in B16F10 cells, 30.38 ± 3.93% of ^64^CuCl_2_ and 27.77 ± 2.91% of ^64^CuCl presented in A375M cells over a 0.5 h incubation period, respectively, indicating that the radioactivity was quickly cleared from cells for both ^64^CuCl_2_ and ^64^CuCl. And only 13.16 ± 1.32% of ^64^CuCl_2_ and 11.13 ± 0.40% of ^64^CuCl were remained in A375M cells at 24 h (Fig. 2B).

### Biodistribution Studies of Mice Bearing B16F10 Tumor

The *in vivo* biodistribution of ^64^CuCl_2_ and ^64^CuCl prepared by ^64^CuCl_2_ with VitC in mice bearing B16F10 tumors was determined at various time points after injection (Table 1). ^64^CuCl displayed similar *in vivo* biodistribution as ^64^CuCl_2_. ^64^CuCl_2_ and ^64^CuCl displayed rapid and similar level of accumulation in the tumor at the early time point (10.81 ± 0.22%ID/g and 11.21 ± 1.06%ID/g at 1 h p.i. for two probes, respectively, *P* > 0.05). At later time points, B16F10 tumor uptake of both ^64^CuCl_2_ and ^64^CuCl slightly increased and reached 12.80 ± 0.53%ID/g and 11.26 ± 1.76%ID/g at 4 h (*P* > 0.05), respectively. B16F10 tumor accumulation of ^64^CuCl_2_ and ^64^CuCl remained 3.14 ± 0.24%ID/g and 3.15 ± 0.23%ID/g, respectively, at 72 h p.i.

**Table 1:** Biodistribution Results of ^64^Cu(II) and ^64^Cu(I) in B16F10 Tumor-Bearing Mice (n = 4).

Organ (%ID/g)	1 h		2 h		4 h		24 h		48 h		72 h	
	Cu(II)	Cu(I)	Cu(II)	Cu(I)	Cu(II)	Cu(I)	Cu(II)	Cu(I)	Cu(II)	Cu(I)	Cu(II)	Cu(I)
Tumor	10.81 ± 0.22	11.21 ± 1.06	11.34 ± 1.79	10.95 ± 0.23	12.80 ± 0.53	11.26 ± 1.76	6.56 ± 0.61	5.95 ± 0.24	3.55 ± 0.14	3.69 ± 0.20	3.24 ± 0.24	3.15 ± 0.23
Blood	3.18 ± 0.16	3.08 ± 0.53	2.67 ± 0.48	2.72 ± 0.35	2.81 ± 0.13	2.53 ± 0.48	1.72 ± 0.16	1.56 ± 0.12	1.45 ± 0.05	1.84 ± 0.28	1.50 ± 0.29	1.49 ± 0.38
Heart	3.95 ± 0.30	3.63 ± 0.29	3.98 ± 0.50	4.50 ± 0.64	4.45 ± 0.65	3.95 ± 0.18	4.11 ± 0.23	4.02 ± 0.46	4.95 ± 0.20	4.96 ± 0.31	4.37 ± 0.61	4.29 ± 0.46
Lungs	12.12 ± 1.18	15.15 ± 0.39	14.50 ± 2.40	13.00 ± 1.25	15.54 ± 1.53	14.04 ± 2.50	9.19 ± 0.78	8.77 ± 0.72	7.52 ± 0.11	6.13 ± 0.10	6.44 ± 0.86	6.33 ± 0.38
Liver	31.66 ± 3.06	36.92 ± 2.47	29.21 ± 3.39	30.27 ± 4.90	29.19 ± 3.61	27.31 ± 4.11	17.88 ± 0.29	18.33 ± 0.98	14.43 ± 1.34	14.25 ± 1.19	13.06 ± 1.43	12.56 ± 1.03
Spleen	3.24 ± 0.78	4.9 ± 0.15	3.32 ± 0.49	5.05 ± 0.21	3.33 ± 1.45	4.98 ± 0.27	3.07 ± 0.24	3.51 ± 0.44	2.81 ± 0.00	3.23 ± 0.90	3.46 ± 0.42	3.36 ± 0.34
Stomach	11.52 ± 1.89	13.38 ± 1.72	14.33 ± 3.24	12.48 ± 2.53	13.90 ± 0.73	10.76 ± 0.99	6.18 ± 0.38	5.80 ± 0.70	4.52 ± 0.34	4.57 ± 0.77	4.02 ± 0.38	4.43 ± 0.25
Pancreas	3.64 ± 0.53	3.63 ± 0.18	5.44 ± 2.71	3.83 ± 0.86	4.09 ± 0.62	3.55 ± 0.40	2.70 ± 0.09	2.60 ± 0.13	2.88 ± 0.10	2.80 ± 0.17	2.46 ± 0.37	2.25 ± 1.30
Intestine	19.89 ± 1.99	24.54 ± 2.39	19.64 ± 3.76	19.63 ± 5.28	16.69 ± 0.60	16.82 ± 0.84	8.12 ± 0.34	7.13 ± 1.09	5.58 ± 0.53	5.24 ± 0.52	2.45 ± 0.19	2.99 ± 0.43
Kidneys	17.06 ± 1.42	17.95 ± 1.38	15.38 ± 0.96	14.37 ± 2.61	15.84 ± 1.85	12.75 ± 2.15	11.47 ± 0.73	10.56 ± 0.43	9.90 ± 1.21	8.77 ± 0.96	11.06 ± 0.86	10.00 ± 0.55
Brain	0.62 ± 0.10	0.68 ± 0.07	0.67 ± 0.08	0.61 ± 0.03	0.78 ± 0.08	0.66 ± 0.13	0.86 ± 0.04	0.77 ± 0.06	0.91 ± 0.04	0.90 ± 0.05	0.90 ± 0.09	0.92 ± 0.08
Skin	2.48 ± 0.46	2.21 ± 0.28	2.40 ± 0.30	2.09 ± 0.53	1.66 ± 0.22	1.21 ± 0.12	0.92 ± 0.08	0.89 ± 0.05	0.88 ± 0.07	0.94 ± 0.19	1.18 ± 0.65	1.14 ± 0.43
Muscle	1.30 ± 0.22	1.00 ± 0.05	1.15 ± 0.05	1.00 ± 0.07	1.09 ± 0.08	1.21 ± 0.65	0.89 ± 0.04	0.80 ± 0.07	1.01 ± 0.08	1.17 ± 0.02	1.03 ± 028	0.70 ± 0.13
Bone	3.13 ± 0.46	3.73 ± 0.86	3.43 ± 0.78	3.00 ± 0.67	2.55 ± 0.25	2.57 ± 0.20	2.30 ± 0.43	2.20 ± 0.05	1.71 ± 0.07	2.17 ± 0.23	1.46 ± 0.25	1.35 ± 0.14

Both ^64^CuCl_2_ and ^64^CuCl displayed rapid blood clearance, as determined by the radioactivity remaining in the blood from 1 h (3.18 ± 0.16%ID/g *vs*. 3.08 ± 0.53%ID/g, *P* > 0.05) to 24 h (1.72 ± 0.16%ID/g *vs*. 1.56 ± 0.12%ID/g, *P* > 0.05). Moreover, ^64^CuCl_2_ and ^64^CuCl also showed low muscle uptake of 1.30 ± 0.22%ID/g *vs*. 1.00 ± 0.05%ID/g at 1 h after injection, which further decreased to 0.89 ± 0.04%ID/g *vs*. 0.80 ± 0.07%ID/g at 24 h p.i. Relative high uptakes of ^64^CuCl_2_ and ^64^CuCl was found in several normal organs such as liver, kidney, lung, stomach and intestine (Table 1). Except the liver and kidney, the accumulation of ^64^CuCl_2_ and ^64^CuCl in other normal organs and tissues obviously decreased at 72 h p.i. The accumulation of ^64^CuCl_2_ and ^64^CuCl in the liver was observed with values of 31.66 ± 3.06%ID/g and 36.92 ± 2.47%ID/g at 1 h p.i. (*P* > 0.05), and decreased to 17.88 ± 0.29%ID/g and 18.33 ± 0.98%ID/g at 24 h p.i., respectively(*P* > 0.05). The accumulation of of ^64^CuCl_2_ and ^64^CuCl in kidneys was also high, with values of 17.06 ± 1.42%ID/g and 17.95 ± 1.38%ID/g at 1 h p.i. (*P* > 0.05), and decreased to 11.47 ± 0.73%ID/g *vs*. 10.56 ± 0.43%ID/g at 24 h p.i. (*P* > 0.05), respectively. These data indicated that ^64^CuCl_2_ and ^64^CuCl were excreted through both the liver and kidneys.

Both ^64^CuCl_2_ and ^64^CuCl displayed moderate to high tumor-to-blood and tumor-to-muscle ratios (Table 2). For example, at 4 h after injection, the tumor-to-blood ratios of ^64^CuCl_2_ and ^64^CuCl were 4.55 ± 0.02 and 4.48 ± 0.39 (*P* > 0.05), and the tumor-to-muscle ratios of ^64^CuCl_2_ and ^64^CuCl were 11.75 ± 0.84 and 10.46 ± 3.26 (*P* > 0.05), respectively.

**Table 2:** Tumor-to-non tumor Ratios of ^64^Cu(II) and ^64^Cu(I) in B16F10 Tumor-Bearing Mice (n = 4).

Ratio	1 h		2 h		4 h		24 h		48 h		72 h	
	Cu(II)	Cu(I)	Cu(II)	Cu(I)	Cu(II)	Cu(I)	Cu(II)	Cu(I)	Cu(II)	Cu(I)	Cu(II)	Cu(I)
Tumor-to-blood	3.40 ± 0.13	3.67 ± 0.32	4.28 ± 0.49	4.19 ± 0.39	4.55 ± 0.02	4.48 ± 0.39	3.85 ± 0.51	3.82 ± 0.41	2.44 ± 0.18	2.02 ± 0.20	2.79 ± 0.42	2.40 ± 0.29
Tumor-to-lung	0.90 ± 0.07	0.74 ± 0.05	0.79 ± 0.16	0.77 ± 0.06	0.83 ± 0.10	0.81 ± 0.10	0.72 ± 0.04	0.68 ± 0.07	0.47 ± 0.01	0.60 ± 0.02	0.63 ± 0.07	0.50 ± 0.01
Tumor-to-liver	0.34 ± 0.03	0.30 ± 0.02	0.39 ± 0.03	0.34 ± 0.06	0.44 ± 0.06	0.41 ± 0.01	0.37 ± 0.04	0.32 ± 0.01	0.25 ± 0.01	0.26 ± 0.01	0.30 ± 0.04	0.25 ± 0.02
Tumor-to-muscle	8.45 ± 1.36	11.22 ± 1.38	9.86 ± 1.31	9.99 ± 0.47	11.75 ± 0.84	10.46 ± 3.26	7.44 ± 1.05	7.43 ± 0.62	3.52 ± 0.40	3.14 ± 0.23	4.11 ± 0.07	4.60 ± 0.67

### Biodistribution Studies of Mice Bearing A375M Tumor

The *in vivo* biodistribution of ^64^CuCl_2_ and ^64^CuCl in mice bearing A375 tumor was determined at 72 h after injection (Table 3). Similar to biodistribution data in mice bearing B16F10 tumor, ^64^CuCl_2_ and ^64^CuCl also showed similar *in vivo* performance. A375M tumor accumulation of ^64^CuCl_2_ and ^64^CuCl was 3.59 ± 0.36%ID/g and 3.44 ± 0.52%ID/g (*P* > 0.05), respectively, at 72 h p.i. The high accumulation of ^64^CuCl_2_ and ^64^CuCl by the liver and kidney was observed, with values of 13.37 ± 1.32%ID/g and 13.29 ± 2.51%ID/g (*P* > 0.05), and 10.34 ± 0.53%ID/g and 8.87 ± 0.60%ID/g (*P* > 0.05), respectively. These data also demonstrated that in mice bearing A375M tumor models, both ^64^CuCl_2_ and ^64^CuCl were cleared through hepatobiliary and renal systems. Moreover, both ^64^CuCl_2_ and ^64^CuCl displayed moderate tumor-to-muscle ratio at 72 h after injection (Table 3). The tumor-to-muscle ratios of ^64^CuCl_2_ and ^64^CuCl were 3.46 ± 1.25 and 2.79 ± 0.58 (*P* > 0.05), respectively.

**Table 3:** Biodistribution Results and Tumor-to-non tumor Ratios of ^64^Cu(II) and ^64^Cu(I) in A375M Tumor-Bearing Mice (n = 4).

Organ (%ID/g)	72 h	
	Cu(II)	Cu(I)
Tumor	3.59 ± 0.36	3.44 ± 0.52
Blood	1.94 ± 0.56	1.90 ± 0.50
Heart	5.86 ± 0.55	6.03 ± 0.48
Lungs	6.15 ± 0.83	5.78 ± 0.60
Liver	13.33 ± 1.27	13.29 ± 2.51
Spleen	3.49 ± 0.15	3.41 ± 0.36
Stomach	5.46 ± 0.76	4.78 ± 1.25
Pancreas	2.84 ± 0.60	2.98 ± 0.22
Intestine	3.82 ± 0.42	3.63 ± 0.65
Kidneys	9.45 ± 0.34	8.87 ± 0.60
Brain	1.00 ± 0.07	0.89 ± 0.14
Skin	1.95 ± 0.36	1.92 ± 0.45
Muscle	1.16 ± 0.30	1.01 ± 0.14
Bone	1.08 ± 0.22	1.04 ± 0.34
**Ratio**		
Tumor-to-blood	1.85 ± 0.27	1.81 ± 0.16
Tumor-to-lung	0.58 ± 0.16	0.59 ± 0.08
Tumor-to-liver	0.27 ± 0.03	0.25 ± 0.10
Tumor-to-muscle	3.09 ± 1.46	2.79 ± 0.58

### Small Animal PET of Mice Bearing B16F10 Tumor

**Figure 3:**
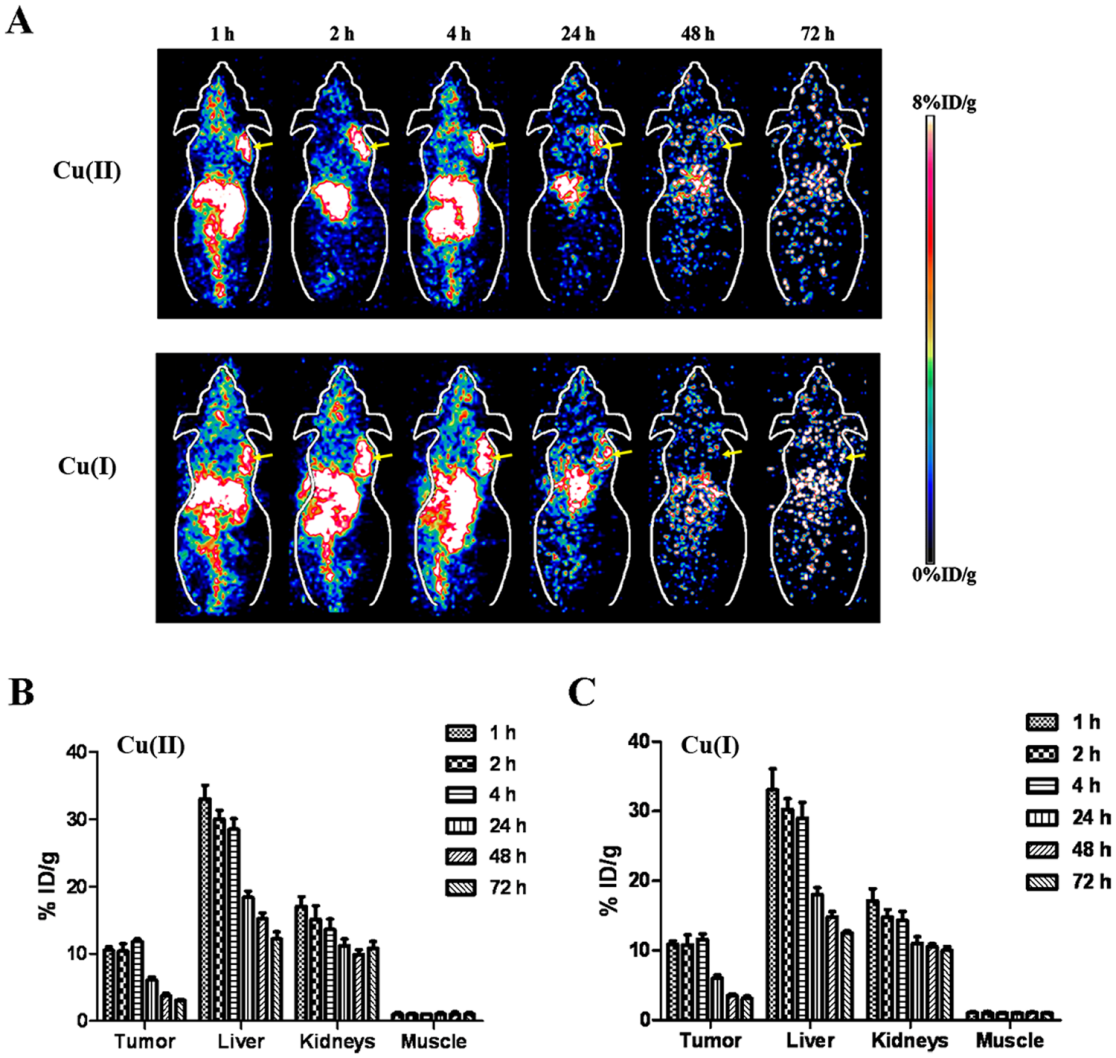
(**A**) Decay-corrected whole-body coronal small-animal PET images of B16F10 tumor-bearing mice at 1, 2, 4, 24, 48, and 72 h after intravenous injection of ^64^Cu(II) and ^64^Cu(I), respectively (Tumors are indicated by arrows). (**B** and **C**) Small-animal PET quantification of tumors and major organs (liver, kidney and muscle) at 1, 2, 4, 24, 48, and 72 h after injection of ^64^Cu(II) and ^64^Cu(I), respectively (n = 4).

Representative coronal and transverse small animal PET images of B16F10 tumor-bearing mice (n = 4) at different time points (1, 2, 4, 24, 48 and 72 h) after injection of ^64^CuCl_2_ or ^64^CuCl were displayed in Fig. 3A. For both probes, the tumors were clearly delineated at 1 h p.i., and persisted to 24 h after injection, which were no longer visible at 48 and 72 h p.i. High liver and kidney uptakes were observed at early time points and beyond, verifying the hepatobiliary and renal clearance route of the two probes. Moreover,other normal organs and tissues displayed relatively low accumulation of ^64^CuCl_2_ or ^64^CuCl at the early time points, and the radioactivity was further decreased after 24 h p.i.

Further quantification analysis (Fig. 3B and C) showed that the tumor uptake of ^64^CuCl_2_ and ^64^CuCl was 11.20 ± 2.18%ID/g and 11.35 ± 2.02%ID/g (*P* > 0.05), respectively, at 1 h p.i., of which values were 3.21 ± 1.72%ID/g and 3.69 ± 1.34%ID/g (*P* > 0.05), respectively, at 48 h p.i. The liver and kidney accumulation of ^64^CuCl_2_ was 35.96 ± 4.04%ID/g and 17.68 ± 3.02%ID/g, respectively, at 1 h p.i., and 11.96 ± 1.11%ID/g and 10.37 ± 1.21%ID/g, respectively, at 72 h p.i. The liver and kidney uptake of ^64^CuCl was similar to that of ^64^CuCl_2_ at various time points, and there was nosignificant difference (*P* > 0.05). Both ^64^CuCl_2_ and ^64^CuCl displayed low muscle uptake after the injection, which was 1.25 ± 0.15%ID/g and 1.05 ± 0.33%ID/g (*P* > 0.05), respectively, at 1 h p.i.

### Small Animal PET Imaging of Mice Bearing A375M Tumor

**Figure 4:**
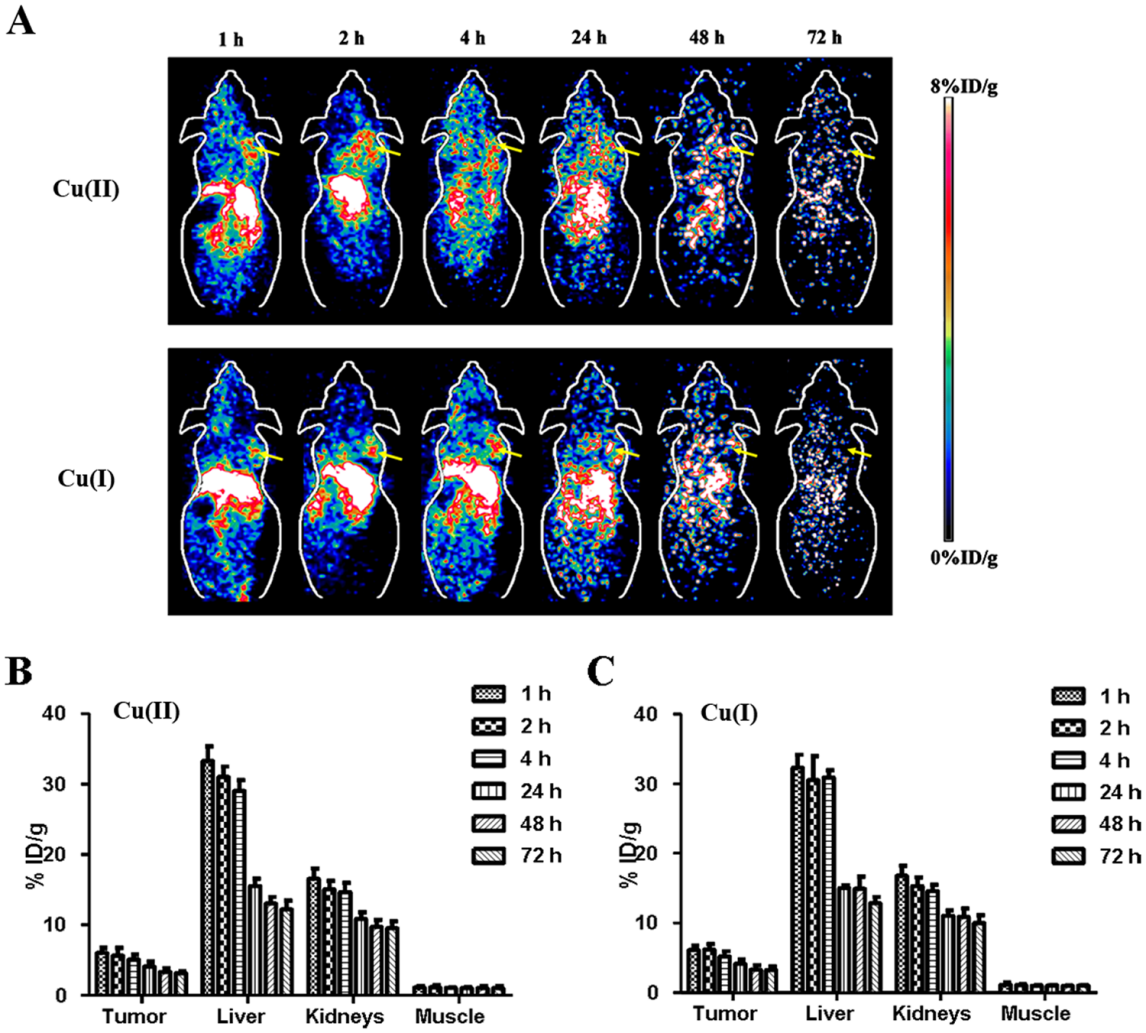
(**A**) Decay-corrected whole-body coronal small-animal PET images of A375M tumor-bearing mice at 1, 2, 4, 24, 48, and 72 h after intravenous injection of ^64^Cu(II) and ^64^Cu(I), respectively (Tumors are indicated by arrows). (**B** and **C**) Small-animal PET quantification of tumors and major organs (liver, kidney and muscle) at 1, 2, 4, 24, 48, and 72 h after injection of ^64^Cu(II) and ^64^Cu(I), respectively (n = 4).

Similarly, Fig. 4A showed representative coronal and transverse small animal PET images of A375M tumor-bearing mice (n = 4) at different time points (1, 2, 4, 24, 48 and 72 h) after injection of ^64^CuCl_2_ or ^64^CuCl. For both ^64^CuCl_2_ and ^64^CuCl, the tumor could be clearly imaged at 1 h p.i. and the high contrast was persisted to 24 h after injection. The tumor was no longer visualized at 48 and 72 h p.i. High liver and kidney uptakes were also observed at all the time points, again indicating the hepatobiliary and renal clearance routes. Moreover, most of the normal organs and tissues also displayed relatively low accumulation of radioactivity after the injection of ^64^CuCl_2_ or ^64^CuCl at the early time points.

The quantification results of small animal PET image analysis were shown in Fig. 4B and C. The tumor uptake of ^64^CuCl_2_ and ^64^CuCl was 6.12 ± 1.38%ID/g and 6.51 ± 1.62%ID/g (*P* > 0.05), respectively, at 1 h p.i., and 3.15 ± 1.22%ID/g and 3.13 ± 1.13%ID/g (*P* > 0.05), respectively, at 48 h p.i. The liver and kidney accumulation of ^64^CuCl_2_ was 32.59 ± 4.20%ID/g and 16.68 ± 2.95%ID/g,respectively, at 1 h p.i., and 12.16 ± 2.01%ID/g and 10.03 ± 1.67%ID/g, respectively, at 72 h p.i. The liver and kidney uptake of ^64^CuCl was similar to that of ^64^CuCl_2_ at 1, 2, 4, 24, 48 and 72 h, and there was no significant difference (*P* > 0.05). Moreover, both ^64^CuCl_2_ and ^64^CuCl displayed low and similarmuscle uptake after the injection, which was around 1%ID/g at 1 h p.i.

### Radiation Absorbed Dose Distribution in Human

The calculated radiation absorbed dose distributions in major organs of a human adult male are shown in Table 4. For both ^64^CuCl_2_ and ^64^CuCl probe, the liver and kidneys showed the highest theoretical radiation absorbed doses; thus, the liver and kidneys would be the dose-limiting organ to carry out cancer-targeted radionuclide therapy. ^64^CuCl_2_ and ^64^CuCl had the similar tumor radiation absorbed dose (1.621 *vs*. 1.808 ID/g·h). They were anticipated to be promising agents for radionuclide therapy of tumors with CTR1 overexpression. Compared with ^64^Cu based probes, ^67^Cu-probes showed higher tumor doses, e.g. ^67^CuCl_2_ (2.695 ID/g·h) and ^67^CuCl (2.971 ID/g·h). However, the ^67^Cu-probes radiodose in normal organs and tissues were also higher than those of ^64^Cu based probes. For example: the radiation absorbed dose by liver for ^67^CuCl_2_ and ^67^CuCl was 1.03 and 0.947 cGy/mCi, respectively, whereas for ^64^CuCl_2_ and ^64^CuCl, it was 0.514 and 0.466 cGy/mCi, respectively.

**Table 4:** Estimated radiation absorbed doses of ^67/64^Cu(II/I) in major organs of an adult male patient based on the biodistribution data obtained from B16F10 tumor-bearing mice.

Target Organ (cGy/mCi)	^64^Cu(II)	^67^Cu(II)	^64^Cu(I)	^67^Cu(I)
Adrenals	0.0388	0.0525	0.0387	0.0517
Brain	0.011	0.0259	0.0106	0.0241
Gallbladder wall	0.0576	0.0763	0.0553	0.0729
Large Intestine Wall	0.244	0.477	0.264	0.488
Small Intestine Wall	0.17	0.331	0.184	0.339
Stomach Wall	0.164	0.303	0.165	0.307
Heart Wall	0.0896	0.204	0.0891	0.207
Kidneys	0.573	1.3	0.66	1.46
Liver	0.514	1.03	0.466	0.947
Lungs	0.0872	0.175	0.0949	0.193
Muscle	0.0104	0.0127	0.0106	0.0128
Pancreas	0.174	0.38	0.197	0.397
Red Marrow	0.0164	0.0219	0.0172	0.0209
Osteogenic Cells	0.02	0.05	0.0216	0.0306
Skin	0.00818	0.0125	0.00865	0.0125
Spleen	0.121	0.251	0.0897	0.189
Testes	0.00687	0.0112	0.00764	0.0114
Thymus	0.0112	0.017	0.0117	0.017
Thyroid	0.00634	0.0111	0.00697	0.0112
Bladder Wall	0.0121	0.0178	0.0132	0.0181
Uterus	0.0185	0.0259	0.02	0.0263
Total Body	0.0329	0.0611	0.0329	0.06
**(ID/g·h)**	^**64**^ **Cu(II)**	^**67**^ **Cu(II)**	^**64**^ **Cu(I)**	^**67**^ **Cu(I)**
Tumor	1.621	2.695	1.808	2.971

## Discussioin

Copper has many important biological roles *in vivo*, such as electron transfer, catalysis, and structural shaping^2^. Many copper-containing compounds are biologically active, and have anti-inflammatory and anti-proliferative properties^24, 25^. On the other hand, Cu can be toxic because of its ability to generate reactive dioxygen species by cycling between Cu(I) and Cu(II) under physiological conditions^26^. Therefore, Cu homeostasis is tightly regulated by the delicate and complex network *in vivo* of influx copper transporter (CTR1), efflux copper transporters (ATP7A and ATP7B), copper chaperons (ATOX1, Cox17, CCS), and other copper binding molecules^27^.

^64^Cu is a cyclotron-produced radionuclide with an intermediate half-life that decays by both β^+^ and β^–^ emission, which makes it suitable for both PET imaging and radionuclide therapy of cancer^28, 29^. Traditionally, ^64^Cu(II) has been widely applied in radiolabeling small molecules, peptides, proteins, antibodies and nanoparticles through various biofunctional chelators, such as 1,4,7-triazacyclononane-1,4,7-triacetic acid (NOTA), 1,4,7,10-tetraazacyclododecane-N, N′, N″, N′″-tetraacetic acid (DOTA) and Triethylenetetramine (TETA), and AmBaSar, which have been proven to display *in vivo* good metabolism in animal models. Some of ^64^Cu(II) labeled probes have been translated into clinical applications, such as successfully using ^64^Cu-DOTATATE for imaging of human neuroendocrine tumors^30^. Recently, with the better understanding the role of CTR1 as a new biomarker for tumor, ^64^CuCl_2_ has been reported to be a novel and promising PET probe for imaging several types of cancers including melanoma, human head and neck cancer, and prostate cancer^12, 22, 31, 32^. However, previous reports indicate that CTR1 is the specific influx copper transporter for Cu(I)^3, 23^. In our previous study, melanoma cell lines B16F10 as well as A375M displayed high level of CTR1 expression, which could be clearly visible by ^64^CuCl_2_ PET imaging^12^. Thus, in this study, both B16F10 and A375M cell lines were continued to use for evaluation of ^64^Cu(I) probe.

It is reported that with the existence of antioxidants VitC or DTT, ^64^Cu(II) can be reduced to ^64^Cu(I)^33–36^. Compared with ^64^Cu(II) uptake by melanoma cell lines, both B16F10 and A375M showed significantly higher ^64^Cu(I) uptake (*P* < 0.05). Wang C *et al*. reported ^64^Cu(II) was also reduced to ^64^Cu(I) under the existence of both DTT and cysteine, which could help the cell uptake of ^64^Cu via CTR1^34^. Interestingly, obvious decreasing cell uptake of copper radioactivity was observed in our study, which may be caused by different tumor cell lines used in different studies. The cell efflux of radioactive copper was further studied in our study. There was small difference of cellular retention of ^64^Cu between ^64^Cu(II) and ^64^Cu(I), and this observation could be attributed to that the cellular efflux of copper was mainly mediated by copper transporters (ATPases)^27, 37, 38^. ATPases, ATP7A and ATP7B, translocate to the cell membrane and function as efflux pumps to excrete copper from cytosol.

B16F10 tumor-bearing mice were well visualized by small animal PET at 1 h after the intravenous administration of ^64^Cu(II) or ^64^Cu(I) via tail vein. Although ^64^Cu was existed in the form of ^64^Cu(I) under the conditions mixed with VitC *in vitro*, the quick changing from unstable Cu(I) to stable Cu(II) could happen because of the dilution of VitC, and next oxidation reaction under physiological conditions *in vivo* ^2, 5^. Therefore, overall, the results of PET image quantitative analysis of ^64^Cu radioactivity in tumor and other normal tissues of ^64^Cu(I) were highly similar to those of ^64^Cu(II). In addition, the biodistribution data for both ^64^Cu(II) and ^64^Cu(I), in general, agreed well with the small animal PET quantification results. Moreover, for A375M tumor-bearing mice, the PET images and biodistribution results of ^64^Cu(II) and ^64^Cu(I) were similar.

Considering the combination therapy with imaging, the radiation absorbed dose distribution of ^64^Cu(I/II) and ^67^Cu(I/II) in a human adult male was analyzed in this study. For ^64/67^Cu(II/I), theoretical radiation absorbed dose distributions in major organs of a human adult male further suggest that the melanoma have high radiation absorbed dose. However, because of the high accumulation of copper in normal organs, such as liver and kidney, and high costs of this kind of radionuclide, ^64/67^Cu could not be recommended for melanoma therapy use unless targeted therapy can be great potentials if with good ideas. It also should be noted that this dose calculation does not mean the exactly same dose distribution results in patient studies. The real patient-specific dosimetry needs to be performed in patient studies.

In conclusion, compared with ^64^Cu(II), ^64^Cu(I) exhibited higher cellular uptake by melanoma, which further testified CTR1 specially influx of Cu(I). However, due to oxidation reaction *in vivo*, no significant difference between ^64^Cu(I) and ^64^Cu(II) was observed through PET images and biodistribution. The *in vivo* stability of ^64^Cu(I) should be further studied to evaluate it as a PET imaging radiotracer.

## Materials and Methods

### Reagents and Cell Culture

^64^CuCl_2_ was purchased from the Department of Medical Physics, University of Wisconsin at Madison (Madison, WI). The pH was adjusted to 7.0 and ^64^CuCl_2_ solution was diluted with phosphate-buffered saline (PBS) buffer (PBS, 0.01 M, pH 7.4). Sodium L-ascorbate (VitC), DL-dithiothreitol (DTT, >99%), cysteine, and copper(II) chloride (CuCl_2_) (97%) were purchased from Sigma-Aldrich (St. Louis, MO).

B16F10 murine melanoma cells and A375M human melanoma cells were obtained from American Type Culture Collection (Manassas, VA), and cultured in Dulbecco’s modified Eagle’s high-glucose medium (DMEM) supplemented with 10% fetal bovine serum (FBS) and 1% penicillin-streptomycin (P/S). The cells were maintained at 37 °C in a humidified 95% air and 5% CO_2_ incubator.

### *In Vitro* Cell uptake and Efflux Studies

Cell uptake and efflux studies were performed on B16F10 and A375M cells, respectively. Briefly, melanoma cells (0.3 × 10^6^ per well, triplicate for each group) were plated in 12-well plates and incubated at 37 °C overnight. The cells were then incubated for various times (0.5, 1, 2 and 4 h) at 37 °C with 37 KBq (1 μCi) ^64^CuCl_2_, 37 KBq (1 μCi) ^64^CuCl [prepared by ^64^CuCl_2_ with VitC (2.5 mM)], 37 KBq (1 μCi) ^64^CuCl [prepared by ^64^CuCl_2_ with and DTT (2.5 mM)], and 37 KBq (1 μCi) ^64^CuCl [prepared by ^64^CuCl_2_ with DTT (2.5 mM) and cysteine (20 mM)] in serum-free medium, respectively. Due to much excess of VitC, DTT or cysteine, Cu(II) could be instantaneously reduced to Cu(I) at the moment of the mixed together and be maintained the reduction statement. Moreover, non-radioactive Cu(II/I) (20 nmol/mL, 0.5 mL per well) was added to block the uptake of ^64^Cu radioactivity in the blocking groups. At designated time points, radioactive medium was aspirated and cells were washed 3 times with ice-cold PBS and lysed with 0.1 M NaOH for 5 min at room temperature. The radioactivity of the cell lysates was counted by a Wallac 1480 automated γ-counter (PerkinElmer, Waltham, MA, USA).

For efflux studies, cells were initially incubated with ^64^CuCl_2_ or ^64^CuCl (prepared by reducing ^64^CuCl_2_ with VitC) for 2 h under the conditions described above, respectively. Then radioactive medium was aspirated and the cells were washed 3 times with PBS buffer. Fresh medium was added and cells were maintained at 37 °C. At different time points (0.5, 1, 2, 4, 16 and 24 h), the supernatant and cell lysate were collected separately and their radioactivity was counted. Cellular retention was calculated by dividing the radioactivity of the cells by the total radioactivity added into the cells at 0 h.

### Subcutaneous Tumor Model

Female C57BL/6 mice and female athymic nude mice (nu/nu) were purchased from Charles River Laboratories (Boston, MA, USA) at 5–6 weeks old and kept under sterile conditions. About 3 × 10^6^ B16F10 and 1 × 10^7^ A375M cells suspended in 100 μL of PBS were implanted subcutaneously into the right shoulders of C57BL/6 mice and nude mice, respectively. Tumors were grown to a size of 0.5–1 cm in diameter (2–4 weeks). All animal experiments were performed under the approval of Stanford University’s Administrative Panel on Laboratory Animal Care (APLAC). All methods were carried out in accordance with relevant guidelines and regulations.

### Small Animal PET Imaging

PET of tumor-bearing mice was performed using a small animal PET scanner (Siemens Invenon). B16F10 tumor-bearing mice (n = 4 for each group) were injected via the tail vein with 2.96–3.33 MBq (80–90 μCi) ^64^CuCl_2_ and 2.96–3.33 MBq (80–90 μCi) ^64^CuCl (prepared by ^64^CuCl_2_ with VitC (2.5 mM)], respectively. At 1, 2, 4, 24, 48 and 72 h post-injection (p.i.), mice were anesthetized with 2% isoflurane (5% for induction and 2% for maintenance in 100% O_2_) for imaging experiments. With the help of a laser beam attached to the scanner, the mice were placed in the prone position and near the center of the field of view of the scanner. Static scans at 24, 48 and 72 h after injection (scanning time, 10 min) and at other time points (scanning time, 5 min) were obtained. The images were reconstructed with two-dimensional ordered-subset expectation maximization (OSEM 2D) algorithm. The method for quantification analysis of small-animal PET images was the same as previously reported^39^. Small Animal PET Imaging and quantification analysis of mice bearing A375M tumors was similar to that of B16F10.

### Biodistribution Studies

Anesthetized B16F10 tumor-bearing mice (n = 4 for each group) were injected with approximately ^64^CuCl_2_ (2.96–3.33 MBq [80–90 μCi]) and ^64^CuCl (prepared by adding 2.96–3.33 MBq [80–90 μCi] ^64^CuCl_2_ with VitC (2.5 mM)], respectively, via the tail vein and sacrificed at different time points from 1 to 72 h p.i. Tumor and normal tissues of interest (blood, muscle, heart, liver, lungs, kidneys, spleen, brain, intestine, skin, stomach, pancreas and so on) were removed and weighed, and their radioactivity levels were measured with a γ-counter. The radioactivity uptake in the tumor and normal tissues was expressed as a percentage of the injected radioactive dose per gram of tissue (%ID/g).

Similar to the biodistribution in B16F10 tumor-bearing mice, A375M tumor-bearing mice (n = 4 for each group) were injected with approximately ^64^CuCl_2_ (2.96–3.33 MBq [80–90 μCi]), and ^64^CuCl_2_ [prepared by adding 2.96–3.33 MBq [80–90 μCi] ^64^CuCl_2_ with Vit C (2.5 mM)], respectively, via the tail vein and sacrificed at 72 h p.i.

### Radiation Absorbed Dose Calculation

To evaluate the possibly clinical application in future, radiation absorbed dose distribution of ^64^Cu(I/II) in a human adult male studied within a 48-h period was calculated based on mice models with B16F10 tumor, using OLINDA/EXM (RADAR, Vanderbilt University, Nashville, TN, USA) software code based on the same % injection dose (ID)/organ^40^. In brief, the accumulated activity in each organ (%ID × time) from 0 to 48 h was obtained by calculating the area under the %ID-time curve. Percent ID in each organ was decay-corrected before the application for accumulated activity calculation. The number of disintegrations in each major organ, (cumulated activity in %ID × h)/100, then was input into OLINDA/EXM software for radiation absorbed dose calculation. Moreover, the radiation absorbed dose distribution of ^67^Cu(I/II) in a human adult male was also analyzed on the basis of ^64^Cu(I/II) data.

### Statistical Analysis

SPSS 18.0 software for Windows (SPSS Inc, Chicago) was used for statistical analysis. The quantitative data were expressed as mean ± SD, and analyzed and compared using the Student *t* test. The 95% confidence level was chosen to determine the significance between groups, with *P* < 0.05 indicating a significant difference.
